# Supplementary material for: PLD3 affects axonal spheroids and network defects in Alzheimer’s disease
Source: Nature. 2022 Nov 30;612(7939):328–37. doi: 10.1038/s41586-022-05491-6 (PMC9729106; doi:10.1038/s41586-022-05491-6)
Supplement: Supplementary file 1 — Supplementary Discussion 1–4 and additional references. Supplementary Discussion 1: estimating the number of axons forming spheroids around individual amyloid plaques. Supplementary Discussion 2: additional analysis of axonal conduction computational modelling results. Supplementary Discussion 3: additional discussion on the impact of PAASs on neural networks involved in memory formation. Supplementary Discussion 4: additional discussion on the impact of PAASs on neural networks involved in memory formation. [file 41586_2022_5491_MOESM1_ESM.pdf]

nature portfolio

https://doi.org/10.1038/s41586-022-05491-6

Supplementary information

PLD3 affects axonal spheroids and network defects in Alzheimer's disease

In the format provided by the authors and unedited

Supplementary information

Supplementary discussion 1: Estimating the number of axons forming spheroids around individual amyloid plaques.

While it is likely that the number of affected axons around plaques varies significantly depending on age, brain region and other factors, we attempted to obtain a ballpark estimation to understand the degree to which individual plaques can disrupt local neural circuits. We used the population distributions of our quantifications of the number of spheroids per axonal segment and the area of individual spheroids as seen in **Figure 2**, to estimate the total PAAS volume in single axon segments (step 1-4 below). The total volume of PAAS per plaque was quantified from ~1000 plaques in the cortex of 6-month-old 5xFAD mice based on LAMP-1 immunohistochemistry. The total PAAS volume per plaque divided by the total PAAS volume per axon segment indicates the number of axons affected per plaque.

Step1: Simulating the number of PAAS in individual axons

As reported in **Extended Data Fig. 1**, the number of spheroids per axon follows a Gaussian distribution with a mean of 4.22 and SD of 1.95. To simulate the number of PAAS from a single axon, we performed a random draw from the distribution using the Norm.inv function in Excel:

=Norm.inv(Rand(), 4.22, 1.95) (function 1)

Next, axons with at least one spheroid were kept for later calculation, using the following code:

=if(result of function 1>0.5, round(result of function 1), “”)
(function 2)

10,000 resulting numbers were kept for later calculation.

Step2: Simulating the volume of individual spheroids

Similar to step 1, we performed a random draw from the Gaussian distribution of the log value of spheroid diameters (**Extended Data Fig. 1**, mean of 0.48 and SD of 0.21). Again, we used the Norm.inv function in Excel:

=Norm.inv(Rand(), 0.48, 0.21) (function 3)

And the resulting values were further transformed to volume:

=4/3 * 3.14 * (0.5*10^(result of function 3))^3 (function 4)

10,000 resulting numbers were kept for later calculation.

Step3: Simulating the total volume of all the spheroids in individual axon segments

To calculate the sum of volumes from multiple spheroids, we first drew a random number from the step1 results and use that number to determine how many random numbers we drew from the step2 results. The total volume can then be calculated by summing these individual spheroids. We achieved this with Excel code:

=Sum(offset(first cell in results of step 2, Randbetween(1:9990), 1, 1, one result from result 1)) (function 5)

10,000 resulting numbers were kept for later calculation.

The distribution of these results (see figure below) shows that the majority of individual PAAS volumes are below 500 μm^3, and occasionally the volume can reach several thousands.

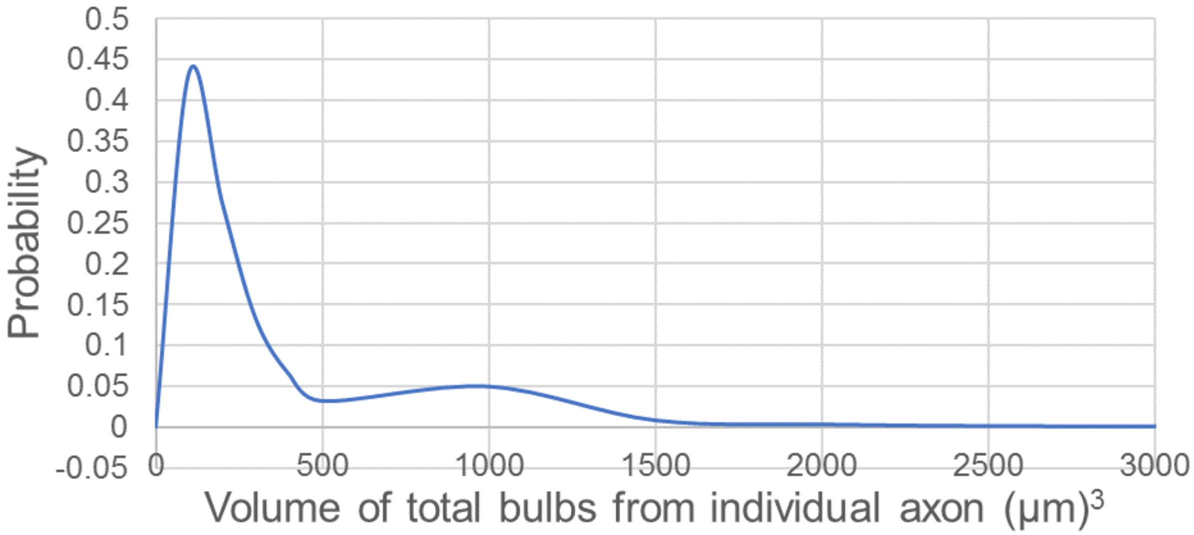

Step4: Simulation of the sum of several axons

By taking a random sample from the results of Step3 as an individual axon, we can then sum several of these samples to represent several axons.

Picking individual axons randomly from the distribution

=offset(first cell in results of step 3, Randbetween(1:9999), 1, 1, 1)
(function 6)

We simulated the summation from 2-200 axons from the results above. The results showed that the total volume of PAAS follows a positive linear correlation with the number of axons, with some noise. The noise almost disappeared when averaging the simulations 10 times, resulting in the following graph:

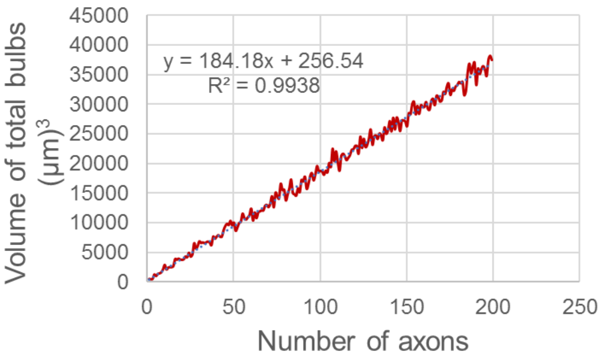

Based on this result, each affected axon segment around plaques adds 184 µm^3 PAAS volume on average to the total halo of PAAS around individual plaques.

<u>Step5:</u> Calculating the number of axons affected around individual amyloid plaques

As described above, we used 1000 PAAS data from 6-month-old 5xFAD mice for this step. We quantified PAAS by measuring the LAMP-1 immunostained area at the center plane of individual amyloid plaques and extrapolated the volume assuming a spherical shape. Then, by dividing the calculated total PAAS halo volume around plaques by 184 (resulting from step 4), we estimated the number of axons underlying this volume. This produces the following plot:

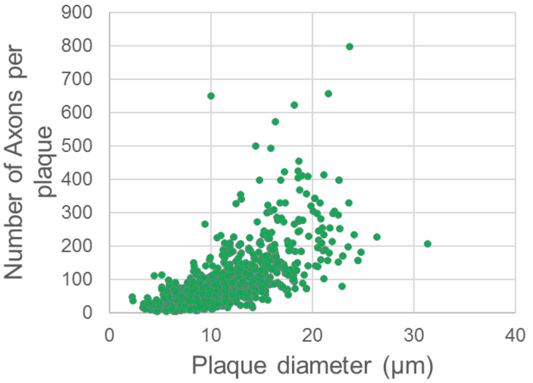

There is a nonlinear, plaque size-dependent increase in the number of affected axons. The results are summarized below, showing the number of affected axons per plaque in relation to plaque size:

	Overall	Plaque Diameter < 10	Plaque Diameter 10-20	Plaque Diameter > 20
Mean	90.12	40.67	115.74	252.5
Standard Error	2.77	1.40	3.82	18.25

Median	63	34	92	220.5
Standard Deviation	88.81	29.39	88.95	126.47
Minimum	2	2	10	78
Maximum	797	265	649	797

Supplementary discussion 2: <u>Additional analysis of axonal conduction computational modeling results</u>

Our computational modeling of action potential propagation along axonal segments near plaques, showed that PAAS were electrically charged with every incoming action potential, and depending on whether the charging depolarizes the membrane potential and reaches the threshold for triggering an action potential, this charging will lead to a conduction delay or block. Such behavior can be fully recapitulated and perhaps more intuitively understood by considering the effect of a simple capacitor charging or discharging in an electric circuit:

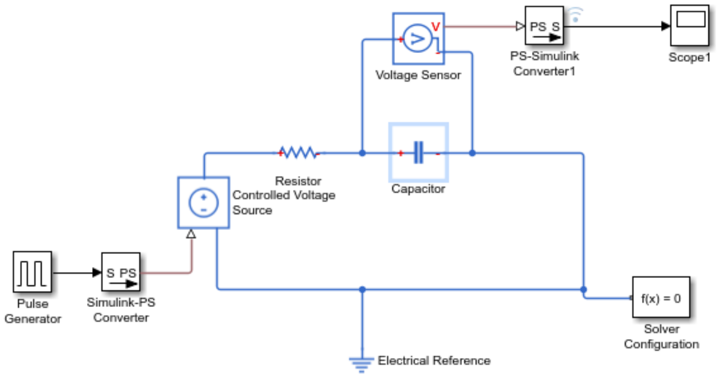

In this simple electric circuit simulated with MATLAB, a voltage source is controlled by a pulse generator to produce a voltage pattern of a simplified action potential. The circuit mimics the action potential propagating through the PAAS. Adjusting the size of the capacitor in this circuit, as shown below, recapitulates the basic findings in our experimental and mathematical modeling resulting from the PAAS effect on axonal conduction. If the pulse generator gives a single pulse, the voltage on the two ends of the capacitor changes over time in the following manner:

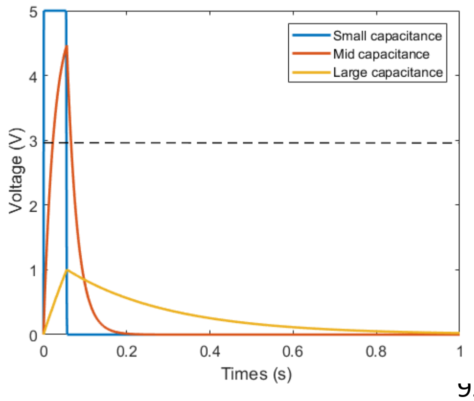

To symbolize the threshold for action potential, we used 3V as an arbitrary line for successful conduction (dashed line). When the capacitance is small, it behaves as a closed wire, and the voltage follows the original pulse (blue line). In the case of middle capacitance (orange line), the slow charging time leads to a delay for the signal to pass through the capacitor. When the capacitance is large, the charge will not reach the threshold (yellow line). These are the categories of results we showed with biophysical modeling of action potential propagation through the axon segment.

To simulate the situation with multiple pulses, we set the pulse generator to produce a 10 Hz signal. In the cases of small and middle capacitance, the capacitor can complete a charge-discharge cycle before the next pulse comes, resulting in 10 successful transmissions. The middle capacitance delays the transmission of each pulse. However, the capacitor produces an interesting behavior in the case of large capacitance. Each pulse charges the capacitor to a certain degree, but the time is too short for the capacitor to discharge completely before the next incoming pulse, leading to a small build-up of voltage with each pulse. In this particular case, it reaches the threshold after 10 pulses, resulting in a delay of action potential propagation of ~1 second, and also reduces the output from 10 pulses to only 1 pulse. This is all consistent with our modeling and experimental results (**Fig. 1, Extended Data Fig. 2**).

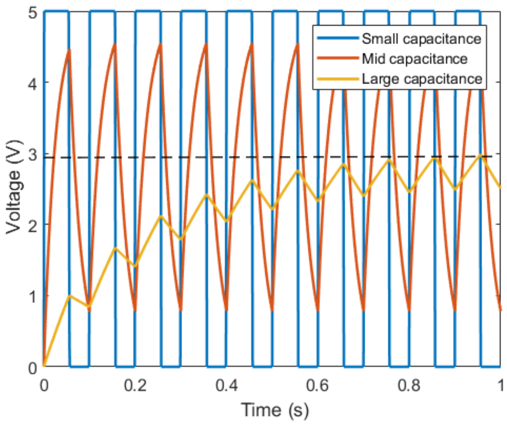

Therefore, it is useful to think of PAAS as an added capacitor in axons. We could use the known mathematical equations for capacitor charge-discharge process to think about the effect of PAAS on axon conduction.

Charge: $Q = CV_a[1 - e^{-\frac{t}{RC}}]$

Discharge: $Q = CV_0 e^{-\frac{t}{RC}}$

Q: total charge; C: capacitance; V: voltage; R: resistance; t: time.

As demonstrated above, these equations can describe the relationship between PAAS capacitance, firing rate and conduction abnormalities.

Importantly, our biophysical model indicates that the capacitance of PAAS is proportional to their size, and thus conduction delay or block should be more prevalent with larger PAAS surface areas.

Supplementary discussion 3: <u>Additional discussion on the impact of PAAS on action potential conduction</u>

To unambiguously demonstrate action potential delays or blockades by PAAS, we implemented ASAP3 voltage sensor to perform in vivo imaging. A single AP induces ~20% change in GCaMP6 intensity[1], while ASAP3 only shows an ~5% intensity decrease[2] in live mouse brain. Because of its slow kinetics (lower decay time) and accumulation of intracellular Ca^{2+}, GCaMP6 has a much larger ∆F/F during AP trains. Due to the negative directionality of change and small amplitude of the signal, ASAP3 is more prone to small vibration artifacts (triggered by heartbeat and breathing), therefore making single axon imaging very challenging. To overcome this issue, we took advantage of the back propagation of antidromic APs. Electrical stimulation was induced on the axon of transcallosal neurons, and two photon line scan imaging was performed on the soma of ASAP3 expressing neurons in the contralateral hemisphere layer V. This strategy allowed us to average multiple pixels on the scan line across the soma (**Fig. 1l**), which greatly improved signal-to-noise ratio and thus eliminated the limitations posed by movement.

One issue that we noticed when comparing interhemispheric conduction times using orthodromic Calcium imaging or antidromic voltage imaging was that the conduction times were not identical (**Fig. 1g, 1p**). However, we do not think that this is related to the sensors themselves because when we compared voltage imaging with ASAP3 with Calcium imaging using GCaMP6 under the same antidromic stimulation conditions., we found that the rise-time of single AP-induced calcium transients were similar to the spike of the ASAP3 transients (**Fig. 1q**), indicating that the Ca^{2+} rise time is a good surrogate of AP spike time.

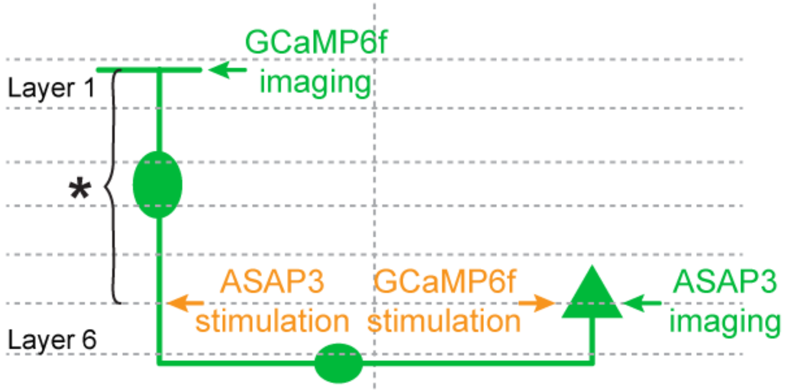

* 500µm thin, unmyelinated axon and spheroids contribute to the extra delay using GCaMP6f

Thus, our explanation for the difference in measurements in interhemispheric conduction with antidromic versus orthodromic stimulations relates to the strategy for stimulation that is required in either condition. Specifically, during orthodromic GCaMP6 calcium imaging, we stimulate layer 5 cortical neuron cell bodies which include many transcallosal projecting cells. Calcium imaging was performed on the contralateral hemisphere superficial axons (~100 um below pial surface). In contrast, during antidromic stimulated voltage imaging with ASAP3, while the stimulation depth was very similar, we had to image the cell bodies of layer V neurons which are hundreds of microns deeper than the superficial axons imaged with GCaMP6. Therefore, the distance traveled by the action potential was much shorter for ASPA3 imaging, which likely explains the reduced conduction time measurements using this sensor, compared to GCaMP6.

Supplementary discussion 4: <u>Additional discussion on the impact of PAAS on neural networks involved in memory formation</u>

We argue that PAAS are particularly detrimental to neural processes that rely on temporally precise long-range coordination among brain regions, such as memory formation. Specifically, during system consolidation, the hippocampus replays the representations of individual memorandums in temporally compressed neural spike sequences[3]. And these sequential replays then guide distributed modification of synaptic connections, closely coupled with network oscillations such as sharp-wave ripples[4] or pontogeniculooccipital waves[5]. Two aspects of this process may be disrupted by PAAS: First, PAAS-mediated conduction delays or blockades could disrupt the faithful propagation of memory-encoding neural sequences in the brain. Similar to PAAS-mediated conduction disruption, experimentally disrupting the precise phase-lock synchronization of neural activities during system consolidation leads to failure of memory

formation[6-8]. In addition, PAAS could further interfere with the synaptic weight modification process, since precise timing of firing in axonal terminals and postsynaptic cells provides pivotal guidance of synaptic plasticity[9,10]. Together, axonal conduction delay and block caused by PAAS may distort the neural processes underlying memory formation, potentially contributing to the anterograde amnesia in AD.

Supplementary References

1. Chen, T. W. *et al.* Ultrasensitive fluorescent proteins for imaging neuronal activity. *Nature* **499**, 295-300, doi:10.1038/nature12354 (2013).
2. Villette, V. *et al.* Ultrafast Two-Photon Imaging of a High-Gain Voltage Indicator in Awake Behaving Mice. *Cell* **179**, 1590-1608 e1523, doi:10.1016/j.cell.2019.11.004 (2019).
3. Buzsaki, G. Hippocampal sharp wave-ripple: A cognitive biomarker for episodic memory and planning. *Hippocampus* **25**, 1073-1188, doi:10.1002/hipo.22488 (2015).
4. Khodagholy, D., Gelinas, J. N. & Buzsaki, G. Learning-enhanced coupling between ripple oscillations in association cortices and hippocampus. *Science* **358**, 369-372, doi:10.1126/science.aan6203 (2017).
5. Ramirez-Villegas, J. F. *et al.* Coupling of hippocampal theta and ripples with pontogeniculooccipital waves. *Nature* **589**, 96-102, doi:10.1038/s41586-020-2914-4 (2021).
6. Wu, C. T., Haggerty, D., Kemere, C. & Ji, D. Hippocampal awake replay in fear memory retrieval. *Nat Neurosci* **20**, 571-580, doi:10.1038/nn.4507 (2017).
7. Jadhav, S. P., Kemere, C., German, P. W. & Frank, L. M. Awake hippocampal sharp-wave ripples support spatial memory. *Science* **336**, 1454-1458, doi:10.1126/science.1217230 (2012).
8. Gridchyn, I., Schoenenberger, P., O'Neill, J. & Csicsvari, J. Assembly-Specific Disruption of Hippocampal Replay Leads to Selective Memory Deficit. *Neuron* **106**, 291-300 e296, doi:10.1016/j.neuron.2020.01.021 (2020).
9. Billings, G. & van Rossum, M. C. Memory retention and spike-timing-dependent plasticity. *J Neurophysiol* **101**, 2775-2788, doi:10.1152/jn.91007.2008 (2009).
10. Dan, Y. & Poo, M. M. Spike timing-dependent plasticity of neural circuits. *Neuron* **44**, 23-30, doi:10.1016/j.neuron.2004.09.007 (2004).
