# Supplementary material for: PLD3 affects axonal spheroids and network defects in Alzheimer’s disease
Source: Nature. 2022 Nov 30;612(7939):328–37. doi: 10.1038/s41586-022-05491-6 (PMC9729106; doi:10.1038/s41586-022-05491-6)
Supplement: Supplementary file 2 — Reporting Summary [file 41586_2022_5491_MOESM2_ESM.pdf]

nature portfolio

Corresponding author(s): Jaime Grutzendler

Last updated by author(s): Sep 23, 2022

Reporting Summary

Nature Portfolio wishes to improve the reproducibility of the work that we publish. This form provides structure for consistency and transparency in reporting. For further information on Nature Portfolio policies, see our Editorial Policies and the Editorial Policy Checklist.

Statistics

For all statistical analyses, confirm that the following items are present in the figure legend, table legend, main text, or Methods section.

n/a	Confirmed	
☐	☒	The exact sample size (*n*) for each experimental group/condition, given as a discrete number and unit of measurement
☐	☒	A statement on whether measurements were taken from distinct samples or whether the same sample was measured repeatedly
☐	☒	The statistical test(s) used AND whether they are one- or two-sided<br>*Only common tests should be described solely by name; describe more complex techniques in the Methods section.*
☐	☒	A description of all covariates tested
☐	☒	A description of any assumptions or corrections, such as tests of normality and adjustment for multiple comparisons
☐	☒	A full description of the statistical parameters including central tendency (e.g. means) or other basic estimates (e.g. regression coefficient) AND variation (e.g. standard deviation) or associated estimates of uncertainty (e.g. confidence intervals)
☐	☒	For null hypothesis testing, the test statistic (e.g. *F*, *t*, *r*) with confidence intervals, effect sizes, degrees of freedom and *P* value noted<br>*Give P values as exact values whenever suitable.*
☒	☐	For Bayesian analysis, information on the choice of priors and Markov chain Monte Carlo settings
☒	☐	For hierarchical and complex designs, identification of the appropriate level for tests and full reporting of outcomes
☐	☒	Estimates of effect sizes (e.g. Cohen's *d*, Pearson's *r*), indicating how they were calculated

Our web collection on statistics for biologists contains articles on many of the points above.

Software and code

Policy information about availability of computer code

Data collection	Confocal images were collected using a Leica SP5 or SP8 system with the associated software. Two-photon images were collected using a Prairie Technology system using the associated Prairie View software (version 5.4), or the Bruker Ultima Investigator multi-photon microscope system. Computational modeling of action potential propagation was carried out in the NEURON environment.
Data analysis	Images were analyzed using customized codes in NIH FIJI and MATLAB software. Statistics were performed with GraphPad Prism (version 7, 8 and 9) software. All codes are available online: https://github.com/PaulYJ/Axon-spheroid.

For manuscripts utilizing custom algorithms or software that are central to the research but not yet described in published literature, software must be made available to editors and reviewers. We strongly encourage code deposition in a community repository (e.g. GitHub). See the Nature Portfolio guidelines for submitting code & software for further information.

Data

Policy information about availability of data

All manuscripts must include a data availability statement. This statement should provide the following information, where applicable:

- Accession codes, unique identifiers, or web links for publicly available datasets
- A description of any restrictions on data availability
- For clinical datasets or third party data, please ensure that the statement adheres to our policy

The authors declare that all derived data supporting the findings of this study are available within the paper and its supplementary information files.

All the custom codes for FIJI and MATLAB used in this study are deposited online: https://github.com/PaulYJ/Axon-spheroid. The scripts are free to the public to view, distribute and use. Our NEURON simulation environment computer code will be made available at ModelDB: http://modeldb.yale.edu/187612. The raw datasets generated and analyzed during the current study are not deposited to a publicly accessible repository due to the large amount of storage required but are available from the corresponding author on reasonable request.

Human research participants

Policy information about studies involving human research participants and Sex and Gender in Research.

Reporting on sex and gender	We obtained postmortem fixed brain tissue from 16 patients with Alzheimer's disease (8 male and 8 female) and 6 with mild cognitive impairment (3 male and 3 female). We did not observe sex-specific effect in our results.
Population characteristics	The detailed demographic information of all the subjects can be found in extended data figure 4. In short we tried our best to balance the sample's age, gender, and ApoE genotype in each group.
Recruitment	The brain tissues were obtained from established brain banks, with standard procedure of informed consent for body donation.
Ethics oversight	The brain tissues were obtained from the Sun Health Research Institute Brain and Body Donation Program, the Mayo Clinic , the University of Washington Alzheimer's Disease Research Center Neuropathology Core and the Alzheimer's Disease Research Center at Washington University at St. Louis (The Knight ADRC Biospecimens Committee). All in complete compliance with the human tissue research use regulation in each organization.

Note that full information on the approval of the study protocol must also be provided in the manuscript.

Field-specific reporting

Please select the one below that is the best fit for your research. If you are not sure, read the appropriate sections before making your selection.

☒ Life sciences ☐ Behavioural & social sciences ☐ Ecological, evolutionary & environmental sciences

For a reference copy of the document with all sections, see nature.com/documents/nr-reporting-summary-flat.pdf

Life sciences study design

All studies must disclose on these points even when the disclosure is negative.

Sample size	For PAAS treatment experiment, the sample size was determined based on previous experiments measuring this pathology (Neuron 90, 724–739, 2016).<br>For calcium imaging of single axons, the sample size was not pre-determined due to the unpredictability of the imaged spheroids. The sample size is justified since the variance within group is small compared to between group differences.<br>For calcium and voltage imaging of interhemispheric axon conduction, a large number of axons were imaged in multiple mice for each group. The observation was highly consistent between mice within each group, indicating that the sample size used was sufficient.
Data exclusions	No data was excluded from the study.
Replication	Three key results of the main paper were replicated.<br>1, PLD3 overexpression was independently repeated once with a different batch of mice.<br>2, PLD3 deletion was independently repeated once with a different batch of mice.<br>3, In vivo calcium imaging has been independently performed by 3 different experimenters, each experimenter has imaged multiple mice from 2019 to 2021. In vivo voltage imaging has been indenpendently performed by 2 different experimenters, each experiment has imaged multiple mice from late 2021 to early 2022.<br>All replications successfully reproduced reported results.
Randomization	In all experiment related to the treatment of PAAS (including quantification of spheroids and calcium/voltage recovery experiments), we used age-matched mice, and then randomly administer the mice with treatment virus or control virus. Sex balance were also taken into consideration.
Blinding	In all experiment related to the treatment of PAAS, images from treatment and control groups were blinded by replacing the file names to random identifiers during analysis via a custom FIJI script. The treatment assignment was only revealed after the measurement of all axon spheroids from the whole experiment was completed.

Reporting for specific materials, systems and methods

We require information from authors about some types of materials, experimental systems and methods used in many studies. Here, indicate whether each material, system or method listed is relevant to your study. If you are not sure if a list item applies to your research, read the appropriate section before selecting a response.

Materials & experimental systems

n/a	Involved in the study
☐	☒ Antibodies
☐	☒ Eukaryotic cell lines
☒	☐ Palaeontology and archaeology
☐	☒ Animals and other organisms
☒	☐ Clinical data
☒	☐ Dual use research of concern

Methods

n/a	Involved in the study
☒	☐ ChIP-seq
☒	☐ Flow cytometry
☒	☐ MRI-based neuroimaging

Antibodies

Antibodies used	1. anti-LAMP1 (DSHB, 1D4B),<br>2. anti-GFP (Aves Labs. Inc. GFP-1020),<br>3. anti-CathepsinD (Abcam, EPR3057Y, ab75852),<br>4. anti-ATP6V0A1 (ThermoFisher Scientific, PA5-54570),<br>5. anti-amyloid precursor protein (ThermoFisher Scientific, LN27, 13-0200),<br>6. anti-PLD3 (Sigma-Aldrich, HPA012800),<br>7. anti-beta amyloid 1-42 (Abcam, ab10148),<br>8. anti-beta amyloid 1-42 (Abcam, mOC98, ab201061),<br>9. anti-MAP2 (Abcam, ab5392),<br>10. anti-Iba1 (Novus Biologicals, NB100-1028),<br>11. anti-S100B (R&D Systems, AF1820),<br>12. anti-Aldh1l1 (NeuroMab, P28037).<br>13. Alexa Fluor dye conjugated secondary antibodies from ThermoFisher.
Validation	All antibodies have been validated in lab through immunofluorescence, to confirm the staining pattern is consistent with that shown on the manufacturer website or by previous literature. In addition, the antibodies have also been validated by the manufacturer as shown by western blot and/or immunofluorescence images on their website.

Eukaryotic cell lines

Policy information about cell lines and Sex and Gender in Research

Cell line source(s)	HEK293T: American Type Culture Collection (ATCC)
Authentication	Authentication was guaranteed by the supplier and authors did not carry out any authentication of the cell lines. The HEK293T cells was used for AAV production only, not for experiments.
Mycoplasma contamination	Cell lines were not tested for mycoplasma.
Commonly misidentified lines<br>(See ICLAC register)	None.

Animals and other research organisms

Policy information about studies involving animals; ARRIVE guidelines recommended for reporting animal research, and Sex and Gender in Research

Laboratory animals	5xFAD (34840-JAX, The Jackson Laboratory) mice were used in this study. Rosa26-LSL-Cas9 (026175, The Jackson Laboratory) mice were crossed with 5xFAD mice for CRSIPR/Cas9-mediated gene deletion.
Wild animals	None.
Reporting on sex	We balanced both sexes in all our experiments to the best of our capability. No sex-specific effects of axonal spheroids on conduction, or modulation of PLD3 on the degree of axonal spheroids were observed in our study.
Field-collected samples	None.
Ethics oversight	Institutional Animal Care & Use Committee (IACUC) at Yale University.

Note that full information on the approval of the study protocol must also be provided in the manuscript.
